# MDGNN: Microbial Drug Prediction Based on Heterogeneous Multi-Attention Graph Neural Network

**DOI:** 10.3389/fmicb.2022.819046

**Published:** 2022-04-07

**Authors:** Jiangsheng Pi, Peishun Jiao, Yang Zhang, Junyi Li

**Affiliations:** ^1^School of Computer Science and Technology, Harbin Institute of Technology (Shenzhen), Shenzhen, China; ^2^College of Science, Harbin Institute of Technology (Shenzhen), Shenzhen, China

**Keywords:** antimicrobial drug prediction, graph convolution networks (GCN), heterogeneous network (Het-Net), representation learning, SARS-CoV-2

## Abstract

Human beings are now facing one of the largest public health crises in history with the outbreak of COVID-19. Traditional drug discovery could not keep peace with newly discovered infectious diseases. The prediction of drug-virus associations not only provides insights into the mechanism of drug–virus interactions, but also guides the screening of potential antiviral drugs. We develop a deep learning algorithm based on the graph convolutional networks (MDGNN) to predict potential antiviral drugs. MDGNN is consisted of new node-level attention and feature-level attention mechanism and shows its effectiveness compared with other comparative algorithms. MDGNN integrates the global information of the graph in the process of information aggregation by introducing the attention at node and feature level to graph convolution. Comparative experiments show that MDGNN achieves state-of-the-art performance with an area under the curve (AUC) of 0.9726 and an area under the PR curve (AUPR) of 0.9112. In this case study, two drugs related to SARS-CoV-2 were successfully predicted and verified by the relevant literature. The data and code are open source and can be accessed from https://github.com/Pijiangsheng/MDGNN.

## Introduction

Microorganisms are the unicellular or multicellular organisms, which include bacteria, archaea, viruses, protists, and fungi (Human Microbiome Project Consortium, 2012; Sommer and Bäckhed, 2013). Microbes sometimes can protect the human body from lethal pathogens, improve metabolism, and strengthen the immune system of the host (Ventura et al., 2009). On the other hand, the imbalance of the microbial community may cause a wide range of human diseases, such as obesity (Zhang et al., 2009), diabetes (Wen et al., 2008), rheumatoid arthritis (Lynch and Pedersen, 2016), and even cancer (Schwabe and Jobin, 2013).

As a novel coronavirus, SARS-CoV-2 has caused an unprecedented public health crisis recently. New variants of SARS-CoV-2 with the enhanced transmissibility are emerging globally. Traditional drug development could not keep pace with threats from the fast-spreading SARS-CoV-2 and its variants, because of the complexity, high cost, and long experiment period of the traditional drug discovery process. The world needs to speed up the drug discovery process for COVID-19.

With the recent development of deep learning, especially the graph neural networks, more and more researchers have begun to try to find solutions based on the deep learning for their biological problems (Shamshirband et al., 2021; Zhang et al., 2021), such as drug interaction identification (Deng et al., 2020; Lin et al., 2020), protein function prediction (Gligorijević et al., 2021), virus classification (Deif et al., 2021), and disease-genes association prediction (Shu et al., 2021), etc. These studies show the potential of graph representation learning in biological questions.

In the research on microorganisms, there is a large amount of known information about the action of microorganisms and drugs, the genetic information of microorganisms, and the molecular formula information of small molecule drugs. We can use calculation-based methods to process these data to predict the possibility of interaction between microorganisms and drugs. This prediction allows us to initially screen out related therapeutic drugs for microorganisms that cause diseases, thereby speeding up the development of specific drugs for related diseases.

For microbial-drug association prediction, there are also several reported methods based on the graph representation algorithms. For example, Zhu et al. (2019) propose a method to predict human microbe-drug association, which is named Human Microbe-Drug Association by KATZ measure (HMDAKATZ). HMDAKATZ predicts possible drug-microbe associations using chemical similarity of drugs based on the Gaussian kernel similarity. Long et al. propose a Heterogeneous Network Embedding Representation framework for Microbe-Drugs Association prediction (HNERMDA) (Long and Luo, 2020). HNERMDA predicts drug-microbe association by heterogeneous graph neural network. Long et al. proposed a graph convolutional network (GCN)-based framework for predicting human microbe-drug associations, named GCNMDA (Long et al., 2020a). GCNMDA predicts drug-microbe association by introducing microbial protein interaction and chemical similarity of drugs. Long et al. propose a framework of heterogeneous graph attention networks to predict the association between drug and microbe (HGATDVA) (Long et al., 2020b). HGATDVA predicts drug-microbe associations by introducing a network of protein interactions between drug targets and microbial hosts. All of these previously reported methods first construct a heterogeneous network with microorganisms and drugs as nodes and then use some network representation methods to get the feature vectors of nodes in the heterogeneous network. For the prediction of the potential association between microorganisms and drugs, a common approach is to first build an action network with a variety of biological information, such as the interaction network between microorganisms and drugs. Then, the graph representation learning algorithm is used to learn node vector representation from the biological interaction network. Finally, the node representation vector obtained by the algorithm is used to predict the probability of potential association between microorganisms and drugs.

Human Microbe-Drug Association by KATZ measure is the first algorithm used to predict potential links between microbes and drugs. In this method, the graph kernel similarity of microorganisms was calculated based on the known conditions to construct the microbial similarity network. Then, the drug similarity network was constructed according to the chemical structure similarity of drugs. By integrating the existing data of microbiota and drug association, a biological network with microbe and drug can be obtained. The KATZ algorithm was then used on this biological network to predict potential associations between microbes and drugs. HNERMDA is a method based on metapath2vec (Dong et al., 2017) to learn the node representation vector of microorganisms and drugs. By constructing an interaction network between microorganisms and drugs, it utilizes metapath2vec to learn their node representation vectors. Then, in the downstream prediction task, the bipartite graph recommendation algorithm with bias is used to predict the potential association between microorganisms and drugs. GCNMDA is a method that uses graph convolutional networks (GCNs) (Kipf and Welling, 2017) to learn node representation in heterogeneous biological networks composed of microorganisms and drugs, obtaining node representation vectors of microorganisms and drugs for the downstream prediction of potential drugs. Host protein information was introduced into the HGATDVA to construct two heterogeneous biological networks: One is a biological network composed of two isomeric nodes of microorganism and drug, and the other is a biological network composed of three isomeric nodes of microorganism, host protein, and drug. During node representation learning, graph attention networks (GAT) (Velickovic et al., 2018) were used to learn network representation of two biological networks, respectively, and two sets of node representation vectors were obtained. The node representation vectors of the two groups of microorganisms and drugs were added to predict the potential association between microorganisms and drugs. In the follow-up prediction of the association between microorganism and drug, the operation is carried out on these node feature vectors. Therefore, a good node feature can make our prediction result more accurate.

In the previously reported studies on the prediction of microbe-drug association, different graph representation learning algorithms are mainly used to improve the prediction performance. With the development of graph neural networks, there are more and more graph representation algorithms with better performance, such as GCN, GAT, heterogeneous graph attention networks (HANs) (Wang et al., 2019), and heterogeneous graph transformer (HGT) (Hu et al., 2020), etc.

In this article, we propose a model incorporating two attention mechanisms into a GCN to enhance the performance of graph characterization algorithms, thereby improving the performance of microbial-drug association prediction. In terms of relevant evaluation indicators, our model is better than the relevant benchmarks. In this case study, we predicted four SARS-CoV-2-related drugs on the relevant dataset through the trained model and verified the effectiveness of two of them in the latest database.

## Materials and Methods

We divide the information in the network into three levels of information. The first is the information inside the node. Generally speaking, entities such as drugs and microbes are abstracted into nodes in the network. To distinguish different nodes, unique features are assigned to nodes, namely, feature vectors of nodes in the network structure. The second is the information between nodes, the edges in the network. Finally, there is edge-to-edge information, the meta-path in the network.

The attention mechanism was first proposed in the field of natural language processing; that is, we can assign different weights to different word vectors. GAT is the earliest method to introduce an attention mechanism in the field of graph neural networks. It assigns different weights to different adjacency features in the stage of information aggregation. This kind of attention is not global attention, but only the attention between first-order neighbor nodes. HAN is an attention-based model for heterogeneous networks, which proposes two attention mechanisms: one is node-level attention, the other is attention between different meta-paths. First, feature vectors from different adjacent points are aggregated on each meta-path by the attention mechanism, and then, feature vectors from each source path are aggregated by assigning different weights to each meta-path. Node-level attention in HAN is still the attention of local nodes, whereas attention between meta-paths is indirect global attention. But this approach relies heavily on setting up the meta-path. HGT is an improved approach to GCN that brings attention to message aggregation by introducing query vectors and key vectors. There are also two attention mechanisms in HGT, namely, attention between local nodes and attention between meta-paths.

However, the existing graph neural networks with attention mechanisms are all based on the local nodes; that is, the attention weight is only allocated between the source node and its neighbors. Due to the limitation of the network structure, the attention information between the source node and its higher-order neighbors is not calculated.

**FIGURE 1:**
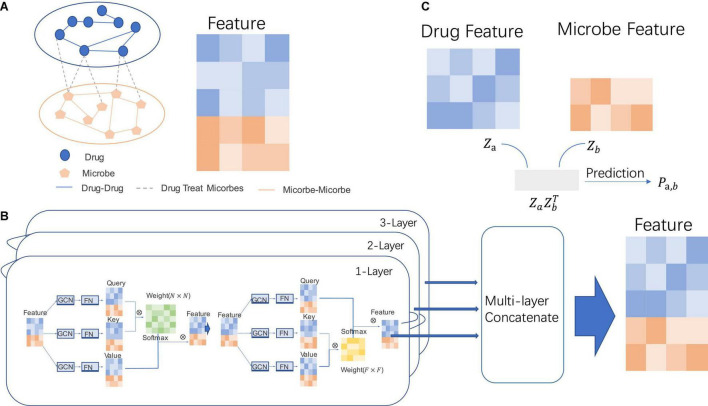
The specific process of MDGNN. **(A)** Microbiome-drug network. The entire network is composed of three subnetworks, namely, the interaction network between drugs and drugs, the interaction network between microorganisms, and the associated network between drugs and microorganisms. **(B)** Graph neural networks of two attentional mechanisms. Through the graph convolution operation on the node feature and the graph structure and the feature projection of the fully connected function, the projection of the three-node features can be obtained, which can be used to calculate the attention weight matrix between nodes and the node feature of attention between feature components, so as to calculate the final node representation features. **(C)** Prediction of potential associations between microorganisms and drugs. Through the inner product of the learned node representations, the prediction score between the drug feature and microbe feature can be obtained, so as to determine whether there is an association between the node pair.

In view of the problems in the above methods, we propose two attention mechanisms, namely, the attention mechanism between all nodes and the attention mechanism between feature components within nodes. Through this new attention-based graph neural network, better node feature vectors for predicting microbial-drug association can be obtained. The whole prediction process is shown in Figure 1. Through node-attention, we can get the attention of one node in the graph to the nodes of the whole graph. Through feature-attention, we can get the weight of each dimension between feature vectors of a node.

The prediction process is to first build a heterogeneous network with drug nodes and microbial nodes. In this network, there are microbial–microbial, microbial–drug, and drug–drug interactions. We mainly predict the potential association between microbial–drug interaction. Then, node-attention and feature-attention mechanisms are used to learn node representation on the network. Finally, after the representation vectors of the two heterogeneous nodes were obtained, they were directly used to predict the link between drugs and microorganisms.

The network representation algorithm is divided into three parts: node-attention, feature-attention, multi-layer feature fusion.

### Node-Attention

Considering the sequence data, in which a single word is used as a data unit and connected together, we can think of sequence data as a special kind of graph structure, which can be regarded as a graph structure in which the in and out degrees of all nodes are 1. Different from GAT’s node-attention mechanism, we also have a weight for higher-order neighbor nodes. Therefore, the advantage of using such global node-attention is that we can aggregate the node information of higher-order neighbors by calculating self-attention, instead of being limited to the structure of the graph to capture the information of other nodes.

Suppose there exists graph G, which can be represented by its adjacency matrix A and node feature matrix X, namely, G = (A, X). For the nodes in graph G, we can calculate their weights and then aggregate the information based on the weights. Different from graph convolution operation, graph convolution operation aggregates information according to the graph structure. When aggregating information according to weight, it can break through the limitation of graph structure and aggregate corresponding information even when there is no edge connection between nodes (refer to Figure 1B).

In this paragraph, we will introduce some commonly used formulas in the following text, such as *GCN*, *Linear*. GCN is a neural network layer that can learn the structure information of graph structure data. The calculation method is shown in Formula (1)


(1) Z=GCN(A,X)=ReLU(ÃXW),

which *X* is the original feature, ReLU is activation function, *W* is the learnable parameter matrix, and $\tilde{A}$ is the adjacency matrix with self-loop of the graph. *Linear* is a fully connected function, and its formula is shown in (2)


(2) $$X^{\prime }=Linear\left(X\right)=WX+b,$$

which *X* is the original feature, and *W and b* are learnable parameter matrix.

Our method is mainly based on the idea that GCN learns the structural information of the network and triplet attention learns the disconnect node interaction information. First, we aggregate node features in GCN, and after learning the structural information of the network, treat all nodes as sequence data and temporarily ignore their structural information, as shown in Formula (3) (σ is non-linear activation function, like ReLU).


(3) GCNLinear=σ(Linear(GCN(G,X)))

By using Formula (1), we can obtain the features of three groups of nodes needed to calculate triplet attention. *Q*_*Node*_, *K*_*Node*_, and *V*_*Node*_. As shown in Formulas (4–6).


(4) $$Q_{Node}=GCNLinear\left(G,X\right)$$

(5) $$K_{Node}=GCNLinear\left(G,X\right)$$

(6) $$V_{Node}=GCNLinear\left(G,X\right)$$

Then, the node weight matrix *W*_*Node*_ (*N* × *N*) is obtained from its inner product, and its row direction is normalized, as shown in Formulas (7, 8).


(7) $$W_{Node}=Q_{Node}⊗K_{Node}^{T}$$

(8) $$w_{i\cdot }=Softmax\left(w_{i\cdot }\right),i\in \left(0,1,2,\ldots ,n\right)$$

Finally, the inner product of weight matrix *W*_*Node*_ (*N* × *N*) and *V*_*Node*_ (*N* × *F*) is integrated to obtain the node feature matrix *X*_*Node*_ (*N* × *F*), as shown in Formula (9).


(9) $$X_{Node}=X+W_{Node}⊗V_{Node}$$

In this process, we model the information of interaction between nodes in the whole network by calculating a node weight matrix *W*_*Node*_ (*N* × *N*). The node weight matrix *W*_*Node*_ (*N* × *N*) is different from the adjacency matrix of the network *A* (*N* × *N*), which can be regarded as the *n* power of the adjacency matrix *A* (*N* × *N*), namely, *W*_*Node*_ (*N* × *N*) = *A^n^* (*N* × *N*) and the *n* varies according to the size of the structure of the network.

### Feature-Attention

The graph can be represented by the node set *V* and the edge set *E*, as well as the node eigenmatrix *X* (*N* × *F*) For any node *N*_*i*_ ∈ *V*, node *N_i_* can be represented by a node feature vector (*f*_1_, *f*_2_, *f*_3_,⋯, *f*_*n*_). For a certain node *N_i_*, we can express the importance of different features by feature weight vectors (*w*_1_, *w*_2_, *w*_3_,⋯, *w*_*n*_), and distribute feature weights by inner product. In other words, for different nodes, there is always some feature components *f_i_*, where *i* ∈ 1, 2, 3, ⋯, *n*. In the dimension of *f_i_*, this node is significantly different from other nodes. For some other feature components *f_j_*, the values of all nodes are almost the same, so we need to give different weight values to these two different feature components. We use a feature component attention weight matrix to model the relationship between feature components within such nodes, as shown in the Figure 1B.

Just as in the calculation of node-attention, three feature vector matrices corresponding to node-features, query, key, and value, are first calculated. The difference lies in that we calculate the weight between node feature components through query and key feature vector matrix, that is, attention weight matrix belonging to feature components, as shown in Formula (10)


(10) $$W_{Feature}=Q_{Feature}^{T}⊗K_{Feature}$$

It should be noted that the node weight matrix is *W*_*Node*_ (*N* × *N*) and the feature component weight matrix is *W*_*Feature*_ (*F* × *F*). After the matrix *W*_*Feature*_ (*F* × *F*) is obtained, the final *W*_*Feature*_ (*F* × *F*) is obtained through the normalization of the column direction, as shown in Formula (10). Then, the final feature vector of nodes is obtained by Formula (11, 12)


(11) $$w_{\cdot \text{j}}=Softmax\left(w_{\cdot \text{j}}\right),j\in \left(0,1,2,\ldots ,n\right)$$

(12) $$X_{Feature}=X+V_{Feature}⊗W_{Feature}$$

### Multi-Layer Feature Concatenates

Generally speaking, GCN can only aggregate information to first-order neighbors of the source node, whereas aggregation to higher-order information requires the number of layers of stacked GCN. For GCN with different layers, the node information represented by GCN is obtained by aggregating the node information within the scope of different graph structures, and these node feature vectors have different structural semantic information. By integrating the node information obtained from these different GCN layers, better results can be obtained for link prediction. For example, in jump-knowledge networks (Xu et al., 2018), node features from different GCN layers are added or spliced as the final node features. It is worth noting that jump-knowledge networks simply add up the node information learned from GCN of different layers and serve as the final node information.

**FIGURE 2:**
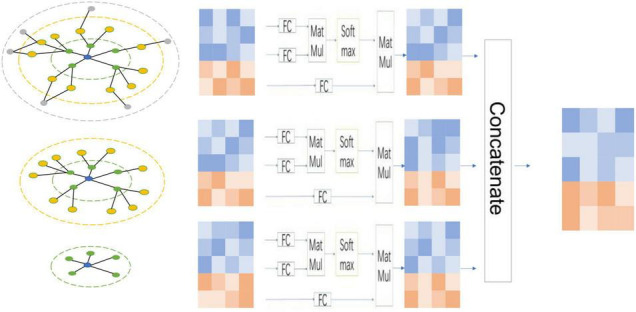
Multi-layer concatenates. For each node-attention and feature-attention, the node information has different structural information. By fusing the features of each layer of nodes based on the two attention mechanisms, it can effectively use these node features integrating different structural information.

Suppose that for graph *G* (*A*, *X*), after *n* layer message aggregation, a list of node features (*X*_1_, *X*_2_, *X*_3_,⋯, *X*_*n*_) will be obtained. The feature vector matrix in this list represents the node features obtained by integrating the substructure information of different graphs. We use triple-based attention to assign different weights to these node features and then fuse them for downstream tasks. We give a schematic diagram of node features obtained by three-layer GNN, as shown in Figure 2. Specifically, for each set of node features, we use the following formulas to calculate,


(13) $$Q_{i}=\sigma \left(Linear\left(X_{i}\right)\right),i\in \left(1,2,\cdots ,n\right)$$

(14) $$K_{i}=\sigma \left(Linear\left(X_{i}\right)\right),i\in \left(1,2,\cdots ,n\right)$$

(15) $$V_{i}=\sigma \left(Linear\left(X_{i}\right)\right),i\in \left(1,2,\cdots ,n\right)$$

After calculating the *Q_i_*, *K_i_*, and *V_i_* corresponding to each group of node features, the final feature vector $X_{i}^{\prime }$ of the group of nodes can be calculated by Formula (16)


(16) $$X_{i}^{\prime }=Softmax\left(Q_{i}⊗K_{i}^{T}\right)⊗V_{i}$$

Finally, by concatenating multiple sets of node features, the final node feature **X**′ can be gain by Formula (17), which can be used to predict the score.


(17) $$X^{\prime }=\left(X_{1}^{\prime }∥X_{2}^{\prime }\cdots ∥X_{n}^{\prime }\right)$$

### Microbial Drug Association Prediction

After getting the final feature vector *X* of the microbe node and the drug node, the prediction score between a certain microbe and the drug node pair can be calculated, that is, the probability of the correlation between the microbe and the drug, as shown in Formula (18)


(18) $$S_{\left(u,v\right)}=Sigmoid\left(X_{u}⊗X_{v}\right)$$

where *X*_*u*_ ∈ ℛ^1×*n*^, *X*_*u*_ ∈ ℛ^1×*n*^, and *Sigmoid* is an activation function.

During the training process, we use binary cross-entropy as our loss function for training, as shown in Formula (19)


(19) $$loss=\sum_{\left(u,v\right)\in pos,neg}BCE\left(S_{\left(u,v\right)},A_{\left(u,v\right)}\right),$$

while *A* is the adjacency matrix, and (*u*, *v*) ∈ *pos* means *A*_(*u*, *v*)_=1, and (*u*, *v*) ∈ *neg* means *A*_(*u*, *v*)_=0.

## Results

### Dataset

In the experiment, we used data coming from three datasets: DrugVirus (Andersen et al., 2020), MDAD (Sun et al., 2018), and aBiofilm (Rajput et al., 2018). We integrated the data of these three datasets after removing duplicate microorganisms and drugs. By calculating the similarity of drug structure, and taking the drug interaction with similarity greater than 0.5 as the relationship between drugs, the drug interaction network is obtained. Similarly, the microbial similarity is calculated through the microbial gene sequence, and the microbial similarity greater than 0.5 is taken as the microbial association to obtain the microbial interaction network. The data used in our experiment are shown in Tables 1, 2.

**TABLE 1:** Data used in this study were obtained by integrating three datasets: DrugVirus, MDAD, and aBiofilm.

Name	Number
Drugs	3,091
Microbes	328
Drug–drug interaction	270,877
Microbe–microbe interaction	467
Drug–microbe interaction	3,900

**TABLE 2:** The statistics for each microbe-drug association dataset.

Datasets	Microbes	Drugs	Associations
MDAD	173	1,373	2,470
aBiofilm	140	1,720	2,884
DrugVirus	95	175	933

### Experiment Result

To verify the effectiveness of our method, we divided the dataset by 5-fold cross-validation of the data related to the known microorganisms a drug and randomly divided the data related to the known microorganisms and drugs into five groups. In each fold experiment, we take turns to select a group of related data as the test set, and the remaining four groups as the training set for training. In addition, because in the real world, it is more common that there is no interaction between microorganisms and drugs. At the same time, to compare the performance of each model in the case of unbalanced positive and negative samples, we set the number of negative samples in the experiment set to four.

In our model, we set that the learning rate in optimization algorithm was 0.001 with Adam optimizer, and other related hyperparameters, such as the number of model layers, feature dimensions, and training times, are described in the ablation experiments. The equipment used in the experiment is Intel(R) Xeon(R) Silver 4114 CPU @ 2.20 GHz, running memory is 128 GB, hard disk storage space is 10TB, and it is equipped with two Tesla P40 GPU with a total memory capacity of 48 GB.

**FIGURE 3:**
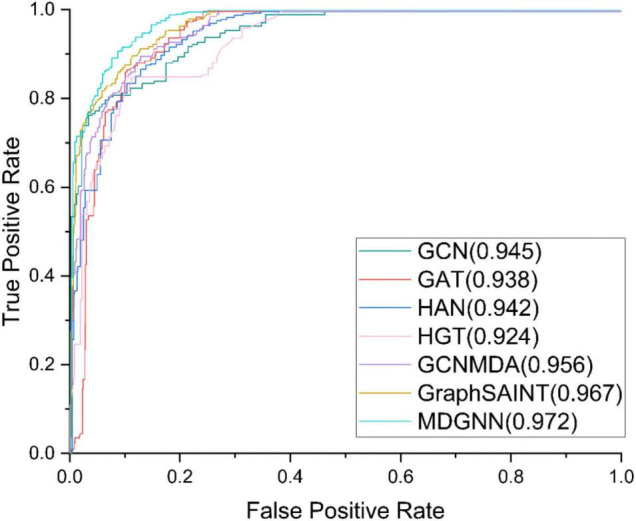
Receiver operating characteristic (ROC) curves of different models under the first-fold verification.

The comparative models we used are GCN, GAT, HAN, HGT, GCNMDA, and GraphSAINT (Zeng et al., 2020). The hyperparameters of the benchmark model are set according to their papers. The experimental results are shown in Table 3. ROC curves of the models are shown in Figure 3.

**TABLE 3:** Comparative experiment of different benchmarks and MDGNN.

Model	AUC	AUPR
GCN	0.9439 (0.0038)	0.8721 (±0.0102)
GAT	0.9385 (0.0057)	0.8479 (0.0139)
HAN	0.9443 (0.0041)	0.8086 (0.0118)
HGT	0.9251 (0.0073)	0.8275 (0.0126)
GCNMDA	0.9541 (0.0036)	0.8796 (0.0103)
GraphSAINT	0.9653 (0.0081)	0.8938 (0.0135)
MDGNN	**0.9721** (0.0053)	**0.9102** (0.0118)

*MDGNN outperforms all baselines including GCN, GAT, HAN, HGT, GCNMDA, and GraphSAINT. The bold value means the best model, and the underlined value means the second-best model.*

Area under the curve (AUC) is an index to measure the sorting performance. It is not sensitive to the balance of positive and negative samples. When the samples are unbalanced, it can also make a reasonable evaluation, which is suitable for measuring the sorting task. The closer of the result is to 1, the better performance it is.

Area under the PR curve (AUPR) is the area value under the curve composed of recall rate and accuracy rate in the prediction results. It is generally used to measure the performance of correct prediction results in the dataset with unbalanced positive and negative samples.

Under a single index, the bold one is the best model, and the underlined one is the second-best model. It can be seen that the performance of our model under AUC evaluation index is ahead of state-of-the-art baseline GraphSAINT. Our model achieves an AUC of 0.9721, better than GraphSAINT, which is 0.9653. Under the evaluation index of AUPR, the performance of our model is significantly ahead of other models. Compared with state-of-the-art baseline GraphSAINT (0.8938), our model (0.9102) has increased by about 1.74%, which is better than GCN (0.8721), GAT (0.8479), HAN (0.8086), HGT (0.8275), and GCNMDA (0.8796).

Through comparative experiments with baseline, it can be seen that our model has achieved a great improvement in performance after calculating the attention between all nodes based on the entire graph. Compared with the model that calculates the attention between 1-hop neighbor nodes, our model is more able to mine the relationship between high-order neighbor nodes, In the association of microbial and drugs, an intuitive idea is if drug A interacts with drug B, and drug A interacts with microorganisms C, then we are likely to be inclined to speculate that drug B and microorganisms C have an interaction. When calculating 1-hop-based attention (such as GAT, HAN, HGT, and GCNMDA), this indirect correlation between drug B and microorganism C is ignored. However, in MDGNN, this indirect correlation will be taken into consideration, and the message will be passed between the nodes B and C through our proposed method, thus improving the prediction performance of the model.

By comparing MDGNN and GCNMDA on three small datasets, we can further confirm our inference. On large dataset (MDAD, aBiofilm), our method is better than GCNMDA, and especially on aBiofilm dataset, our method can be nearly a percentage point higher than GCNMDA. This dataset is the most data in these three datasets. On the smallest dataset (DrugVirus), our method (AUC:0.8737, AUPR:0.8943) is inferior to GCNMDA (AUC:0.8986, AUPR:0.9038).

According to the results in Table 4, it can be seen that when the size of dataset grows, the number of indirect associations (like the relationship between B and C mentioned above) in the dataset will increase accordingly. This means that on large dataset, our method can learn more information about potential associations, and many of our final predictions of the association between microorganisms and drugs are inferred based on this potential association information.

**TABLE 4:** Compare the 5-fold crossover experimental results of MDGNN and GCNMDA on three small datasets (MDAD, aBiofilm, and DrugVirus).

Methods	MDAD		aBiofilm		DrugVirus	
	AUC	AUPR	AUC	AUPR	AUC	AUPR
GCNMDA	0.9423 (0.0105)	0.9376 (0.0115)	0.9517 (0.0035)	0.9488 (0.0031)	0.8986 (0.0305)	0.9038 (0.0372)
MDGNN	0.9457 (0.0083)	0.9431 (0.0102)	0.9608 (0.0054)	0.9566 (0.0084)	0.8737 (0.0167)	0.8904 (0.0212)

It can be seen that the calculation of the two kinds of attention brings stronger fitting ability to the model. Moreover, this powerful fitting ability allows our model to learn more structural information every time it performs gradient descent, so as to converge more quickly during the training process.

**FIGURE 4:**
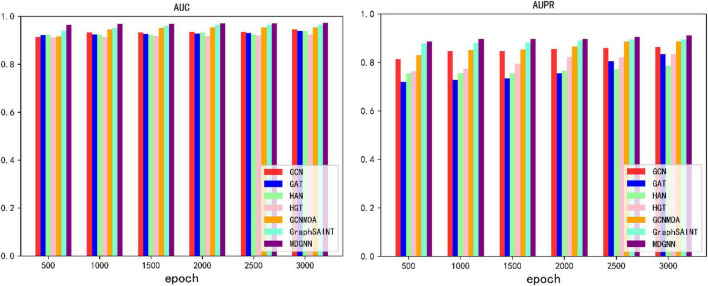
AUC, AUPR values of different numbers of epochs.

During the training process, we found that the optimization of these baseline training is extremely slow, and our model converges fast, so we train the model under different epochs settings to compare the effect of model training. When different numbers of epochs were set, the results obtained by each model are shown in Figure 4.

As can be seen from the experimental results, our model can converge to the optimal value within a very short training period. Under the same epoch value, our model has greatly improved compared with other models. MDGNN requires less than 500 epochs to make the AUC converge to above 0.96, while other comparison models fail to exceed 0.95 after 3,000 epochs.

Through ablation experiments, we can analyze the role of each module. In the experiment, we analyze the function of each module by setting a model with different number of blocks. The specific ablation experimental results are shown in Table 5.

**TABLE 5:** Ablation experiments on modules of feature-attention and multi-layer feature.

Multi-layer	Feature-attention	Layer	AUC	AUPR
w/o Multi-layer	w/o Feature-attention	3 layers	0.9621	0.8891
		4 layers	0.9614	0.8816
		5 layers	0.9652	0.8909
	Feature-attention	3 layers	0.9637	0.8896
		4 layers	0.9640	0.8909
		5 layers	0.9694	0.9034
Multi-layer	w/o Feature-attention	3 layers	0.8696	0.7362
		4 layers	0.8760	0.7264
		5 layers	0.8756	0.7410
	Feature-attention	3 layers	0.9706	0.8982
		4 layers	0.9711	0.9097
		5 layers	**0.9726**	0.9112

*The bold value means the best model.*

As can be seen from Table 4, when both modules are used, the performance is the best. Specifically, when the node information integrated with node-attention is directly aggregated through multi-layer module, the model will produce negative optimization. The reason may be that after removing the feature-attention, the calculation in the multi-layer module is performed directly on the node vector that incorporates the node-attention, which will cause the decoupling of the attention calculated in the node-attention, which results in a decrease in the result.

**FIGURE 5:**
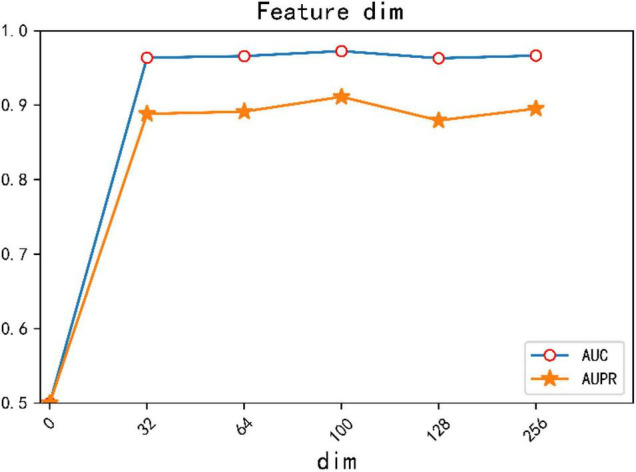
AUC, AUPR values of different feature dimensions.

In addition, we conducted comparative experiments on the dimensions of different feature vectors and verified that the best experimental results were obtained when the dimension of feature vector was set to 100. The result is shown in Figure 5.

### Case Study

In case study, we use the deduplicated datasets, which contains the SARS-CoV-2-related data from the DrugVirus dataset. We save the trained model parameters and use them to make predictions on the entire dataset. The parameters of the model are trained on the randomly divided training set, selected, and saved according to the results on the test set.

We load the trained model and then input the entire dataset into the model to obtain the feature vector of microorganisms and the feature vector of drugs. The corresponding microbial drug association score is obtained by inner product of the feature vector of microbe and the feature vector of drug.

Taking SARS-CoV-2 as an example, we predicted the drugs that may treat the virus and took out the 10 drugs with the greatest possibility. The results are shown in Table 6.

**TABLE 6:** Predicted drugs that can treat SARS-CoV-2 (negative means that the drug is not associated with SARS in our dataset).

Predicted drugs	Prediction score
Mefloquine	0.9361
Darunavir (negative)	0.9177
Nelfinavir	0.9096
Azithromycin(negative)	0.8904
Vancomycin	0.8731
Dicinnamyl (negative)	0.8685
Niclosamide	0.8663
Chitosan (negative)	0.8529
Chlorpromazine	0.8467
Ribavirin	0.8406

Among the ten drugs that we predicted to treat SARS-CoV-2, four drugs were not associated with SARS-CoV-2 in our dataset, but our model predicted that these four drugs had a high potential to treat SARS-CoV-2. Through searching PubChem database, we found that two of the four drugs can indeed treat SARS-CoV-2. Darunavir is an antiretroviral protease inhibitor that is used in the therapy and prevention of human immunodeficiency virus (HIV) infection and the acquired immunodeficiency syndrome (AIDS) (Deeks, 2018). In our dataset, there is indeed an association between Darunavir and HIV, but there is no association between Darunavir and SARS-CoV-2 (Costanzo et al., 2020). This real association does not exist in our dataset, and we can predict this association through the dataset. Similarly, Azithromycin is a drug that can treat SARS-CoV-2 (Rosenberg et al., 2020). However, there is no association between Azithromycin and SARS-CoV-2 in our dataset where Azithromycin is only associated with Hepatitis C virus and HIV. In addition, our model successfully predicts the potential association between Azithromycin and SARS-CoV-2.

## Conclusion

With the rapid development of deep learning, there are many deep learning methods reported for drug development. For example, Beck et al. identified commercially available drugs to treat viral proteins using a pretrained deep learning-based drug target interaction model. Their results showed that drugs used to treat HIV might be effective against SARS-CoV-2 (Beck et al., 2020). Joshi et al. (2020) used deep learning methods to predict the structural formula of chemical molecules and predict potential drugs for SARS-CoV-2. A total of 39 potential drugs for SARS-CoV-2 were predicted based on the CHEMBL dataset.

The rapid spread of SARS-COV-2 and its variants have resulted a serious public health crisis. How to develop a specific drug quickly to tackle SARS-CoV-2 and its variants is an urgent problem. We propose a novel attentional mechanism-based graph neural network framework for learning network node representation and prove that our framework is superior to other state-of-the-art methods, which includes GCN, GAT, HAN, and HGT, etc. In addition, through a large number of drug and microbial data, we have screened potential drugs for the treatment of SARS-CoV-2, most of which are known to treat SARS-CoV-2.

## Data Availability Statement

The original contributions presented in the study are included in the article/supplementary material, further inquiries can be directed to the corresponding authors.

## Author Contributions

JP and JL designed the study, performed bioinformatics analysis, and drafted the manuscript. JL conceived the study and coordination and drafted the manuscript. All authors participated in the revision of the manuscript and read and approved the final manuscript.

## Conflict of Interest

The authors declare that the research was conducted in the absence of any commercial or financial relationships that could be construed as a potential conflict of interest.

## Publisher’s Note

All claims expressed in this article are solely those of the authors and do not necessarily represent those of their affiliated organizations, or those of the publisher, the editors and the reviewers. Any product that may be evaluated in this article, or claim that may be made by its manufacturer, is not guaranteed or endorsed by the publisher.
